# ﻿ *Hydrangeamarunoi* (Hydrangeaceae), a new species from Osumi Peninsula, southern Japan

**DOI:** 10.3897/phytokeys.211.89452

**Published:** 2022-10-11

**Authors:** Shuichiro Tagane, Shinji Fujii, Shun K. Hirota, Akiyo Naiki, Tetsukazu Yahara

**Affiliations:** 1 The Kagoshima University Museum, Kagoshima University, 1-21-30 Korimoto, Kagoshima, 890–0065, Japan Kagoshima University Kagoshima Japan; 2 Department of Field Ecology, University of Human Environments, Okazaki, Aichi, 444–3505, Japan University of Human Environments Okazaki Japan; 3 Field Science Center, Graduate School of Agricultural Science, Tohoku University, 232–3 Yomogida, Narukoonsen, Osaki, Miyagi, 989–6711, Japan Tohoku University Osaki Japan; 4 Iriomote Station, Tropical Biosphere Research Center, University of the Ryukyus, 870 Uehara, Taketomi-cho, Yaeyama-gun, Okinawa, 907–1541, Japan University of the Ryukyus Okinawa Japan; 5 Kyushu Open University, 744 Motooka, Fukuoka, 819–0395, Japan Kyushu Open University Fukuoka Japan

**Keywords:** *Cardiandra*, endemic species, flora, MIG-seq, plant taxonomy, Saxifragales

## Abstract

*Hydrangeamarunoi* Tagane & S. Fujii, from the Kimotsuki Mountains in the Ohsumi Peninsula, southern Japan, is described and illustrated. It is morphologically similar to *H.alternifolia* in having three-petaloid calyx lobes in marginal flowers, but is distinguished by the larger stamen number, and longer styles and seeds. Multiplex ISSR genotyping by sequencing (MIG-seq) demonstrated that the new species is monophyletic and closely related to *H.amamiohsimensis* and *H.moellendorffii* rather than *H.alternifolia*.

## ﻿Introduction

*Hydrangea* L., including approximately 270 natural species (De Smet et al. 2015) and four widely cultivated species (Fulcher et al. 2016), is a well-known genus in Hydrangeaceae. Based on phylogenetic analysis, De Smet et al. (2015) proposed a broad circumscription of *Hydrangea* comprising approximately 200 species distributed across East and Southeast Asia and the Americas. Most *Hydrangea* species are shrubs or lianas. However, the species of Hydrangea L. sect. Cardiandra (Siebold & Zucc.) Y.De Smet & Samain are herbs that have been treated as members of the genus *Cardiandra* (Ohba 1985a, b, 2001; Wei and Bartholomew 2001; De Smet et al. 2015). In the current broad circumscription of *Hydrangea*, it is treated as a section of the genus *Hydrangea*, which additionally also includes eight groups previously known as genera i.e. *Broussaisia* Gaudich, *Decumaria* L., *Deinanthe* Maxim., *Hydrangeas.str.*, and *Pileostegia* Hook. f. & Thomson, *Platycrater* Siebold & Zucc., and *Schizophragma* Siebold & Zucc. In the phylogeny by De Smet et al. (2015), Hydrangeasect.Cardiandra is monophyletic and a sister to the sect. Deinanthe, which comprises two known herbaceous species from China to Japan.

In Hydrangeasect.Cardiandra, four species, *H.alternifolia* L., *H.amamiohsimensis* (Koidz.) Y. De Smet et Granados, *H.moellendorffii* Hance, and *H.densifolia* (C. F. Wei) Y. De Smet & Granados are known from Japan, Taiwan and China (Ohba 1985a, b, 2001; Wei and Bartholomew 2001; Ohashi 2017), all but *H.densifolia* are known from Japan.

During our floristic survey in Kagoshima Prefecture, southern Japan in 2021, we collected an unknown flowering species of HydrangeasectCardiandra. It is similar to *H.alternifolia* in appearance, but it differs from it in its habitat and some floral characters. To clarify the relationship between the unknown species and the other species of Hydrangeasect.Cardiandra in Japan, we examined the phylogenetic relationships of 52 samples of *H.alternifolia*, *H.amamiohsimensis*, *H.moellendorffii*, and the unknown species using multiplex ISSR genotyping by sequencing (MIG-seq, Suyama and Matsuki 2015) and compared this with our observations of morphological characteristics. Multiplexed inter-simple sequence repeats (ISSR) genotyping by sequencing (MIG-seq) is a technique used to obtain many single nucleotide polymorphisms (SNPs) throughout a genome, which is valuable for determining molecular phylogenetic trees. It has been successfully applied to resolve the taxonomy of closely related taxa, including in *Hydrangea* (Hirota et al. 2022). Based on the phylogenetic hypotheses resulting from MIG-seq analysis and subsequent morphological observations, we describe *Hydrangeamarunoi*, sp. nov.

## ﻿Materials and methods

### ﻿Morphological observation and assessment of conservation status

To assess the novelty of the unknown species, we consulted the taxonomic literature (Ohba 1985a, b, 2001; Wei and Bartholomew 2001; Ohashi 2017) and herbarium specimens at FU, KAG, KAP, and TI, as well as the digitized specimen images of FKSE, TRPM, and those available at the Shimane Nature Museum of Mt. Sanbe available on the web (Digital herbarium of Shimane University Faculty of Life and Environment Sciences http://tayousei.life.shimane-u.ac.jp/harbarium/).

The conservation status was calculated following the IUCN Red List categories and criteria v3.1 (IUCN 2012) and IUCN guideline (IUCN 2019). The Extent of Occurrence (**EOO**) and Area of Occupancy (**AOO**) were calculated using the GeoCAT software (Bachman et al. 2011).

### ﻿Taxon sampling for phylogenetic analysis

**Figure 1.**
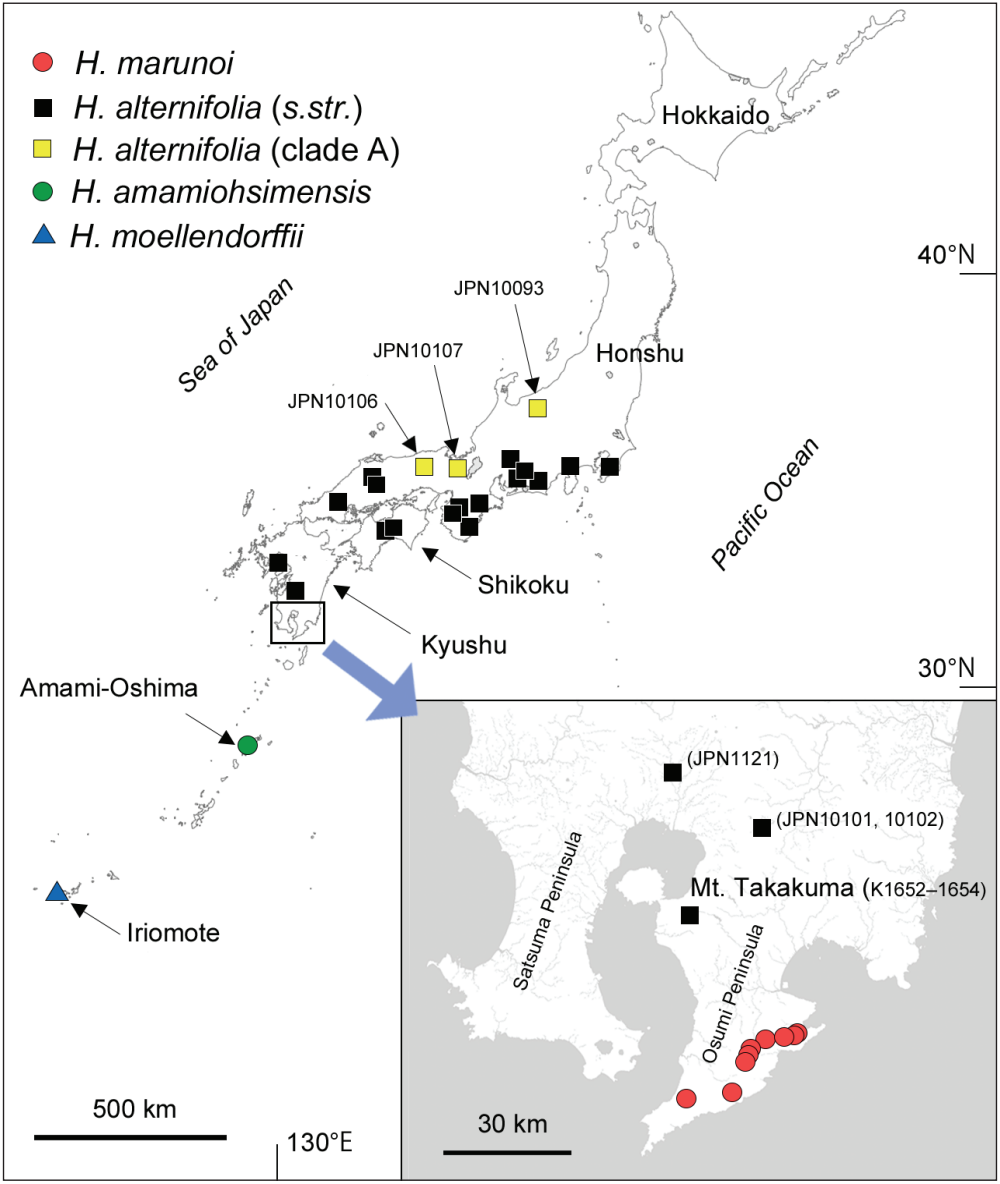
Collection localities of the four Hydrangeasect.Cardiandra species in this study.

To perform the phylogenetic analysis, 52 samples of the Hydrangeasect.Cardiandra were gathered from both our field surveys in Japan and herbarium specimens deposited at the Kagoshima University Museum (KAG): 10 *H.marunoi* samples, two *H.amamiohsimensis* samples, four *H.moellendorffii* samples, and 36 *H.alternifolia* samples (Suppl. material 1: Table S1; Fig. 1). Additionally, one sample of *H.bifida* (Maxim) Y.De Smet & C.Granados of Hydrangeasect.Deinanthe was used as the outgroup. During the field survey, a small piece of leaf was cut, placed in a tea bag, and dried with silica gel in a zip-lock bag.

### ﻿MIG-seq analysis

Total DNA was extracted from dried leaves using the cetyl trimethylammonium bromide (CTAB) method (Doyle and Doyle 1990). For *de novo* SNP detection, MIG-seq (Suyama and Matsuki 2015) was performed according to the protocol described by Suyama et al. (2022). To prepare the MIG-seq library, a two-step PCR amplification process was performed: ISSR regions were amplified using the first PCR, and Illumina sequencing adaptors and indices were added to the first PCR products during the second PCR. Sequencing was performed on an Illumina MiSeq platform (Illumina, San Diego, CA, USA) using a MiSeq Reagent Kit v3 (150 cycles; Illumina). We skipped the sequencing of the first 17 bases of reads 1 and 2 (SSR primer regions and anchors) using “DarkCycle.” Low-quality and extremely short reads containing adapter sequences were removed using Trimmomatic 0.39 (Bolger et al. 2014). The Stacks 2.60 pipeline (Rochette et al. 2019) was used for *de novo* SNP genotyping with the following parameters: the minimum depth of coverage required to create a stack (m) = 3, the maximum distance between stacks (M) = 2, and the maximum mismatches between loci when building the catalog (n) = 2. Three criteria were used for the SNP filtering. First, any SNP site where one of the two alleles had less than three counts was filtered out owing to the difficulty in distinguishing polymorphisms from sequencing errors when the minor allele count of SNPs is extremely low (Roesti et al. 2012). Second, SNPs with high heterozygosity (Ho ≥ 0.6) were removed because excess heterozygosity may have resulted from artifactual loci built from several paralogous genomic regions. Third, SNPs with a genotyping rate of < 30% were eliminated. Using the third criterion, the SNPs retained by at least 16 samples were included in the SNP dataset.

Maximum likelihood phylogeny based on SNPs was inferred using the RAxML 8.2.10 software (Stamatakis 2014). We used a GTRCAT model with an ascertainment bias correction using the Lewis method and performed 1,000 replicates of parallelized tree search bootstrapping.

## ﻿Results

**Figure 2.**
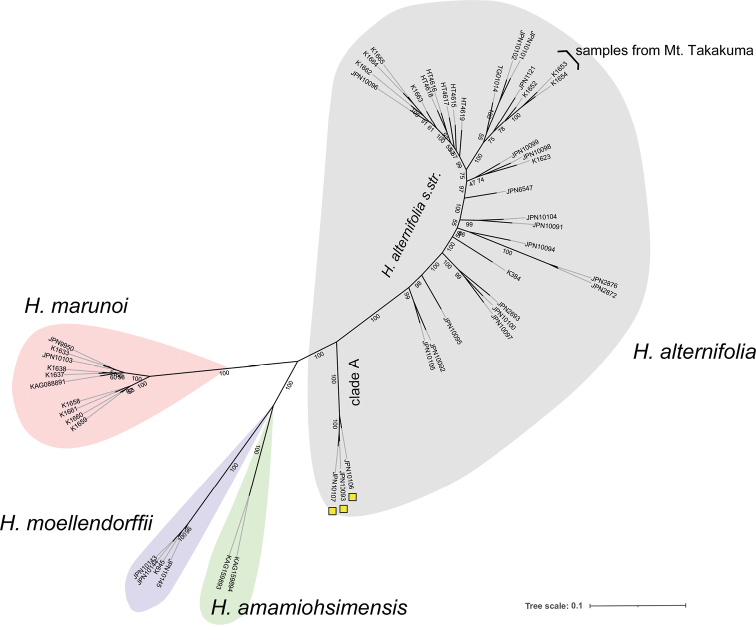
Molecular phylogenetic tree using MIG-seq data of 52 samples of Hydrangeasect.Cardiandra. Bootstrap values are shown on the internodes.

Among the 17,753,114 raw reads (334,964 ± 34,812 reads per sample) obtained, 13,254,044 reads (250,076 ± 28,521 reads per sample) remained after quality control. After *de novo* SNP detection and filtering, 1875 loci and 4506 SNPs were identified. *Hydrangeabifida* (JPN4970) was removed from the SNP dataset because of its high proportion of missing data (0.982). The ten *H.marunoi* samples were monophyletic and formed two geographically defined groups; populations from Mt. Nokubi (K1658–1661) and that from the Oda River (JPN9950, JPN10103, K1633, K1637, K1638, KAG088891) (Fig. 2). *Hydrangeamarunoi* was sister to a clade that included *H.amamiohsimensis* and *H.moellendorffii*. *Hydrangeamarunoi*, *H.amamiohsimensis*, and *H.moellendorffii* were all supported as monophyletic by bootstrap values of 100%. *Hydrangeaalternifolia* was sister to a clade that included these three species. Three samples of *H.alternifolia* collected from Tottori, Kyoto, and Niigata prefectures (JPN10093, 10106, 10107, respectively, Fig. 1), located on the Sea of Japan (western) side of Honshu Island, were clustered at the base of *H.alternifolia*; this clade, supported by a 100% bootstrap value, was closer to the clade including *H.marunoi*, *H.amamiohsimensis*, and *H.moellendorffii* (Fig. 2, clade A).

### ﻿Taxonomic treatment

#### ﻿ Hydrangea marunoi

Taxon classificationPlantaeCornalesHydrangeaceae

Tagane & S.Fujii sp. nov.

BAB9FAC7-F979-5AAE-85D9-BEE84AD321C4

urn:lsid:ipni.org:names:77306481-1

Figs 3 , 4

##### Diagnosis.

*Hydrangeamarunoi* is similar to *H.alternifolia* in having three-petaloid calyx lobes in marginal flowers but is distinguished by its higher number of stamens (28–32 stamens in *H.marunoi* vs.15–26 stamens in *H.alternifolia*), longer styles (1.2–1.8 mm long in fruiting vs. to 1.2 mm long), and longer seeds (1.2–1.5 mm long vs. 0.7–1 mm long).

##### Type.

Japan. Kagoshima Prefecture, Kimotsuki Town, along the Oda River, 8 Aug. 2021, *S. Tagane K1637* (holotype: KAG 153198!; isotype: KYO!).

##### Description.

Rhizomatous perennial herb, 31–103 cm tall. Stems green *in vivo*, grayish-brown when dry, 3.5–6 mm in diameter near the base, puberulous when young, and subsequently glabrous. Leaves alternate, 7–11 per stem, petiolate; blades ovate, ovate-elliptic, elliptic-oblong, obovate-elliptic, (6.5–)10–28.4 × (2.5–)3.3–10.5 cm, chartaceous, sparsely pubescent to subglabrous on both surfaces, grayish-green adaxially, light pale green abaxially, apex acuminate, acumen to 2.5 cm long, base cuneate, decurrent, margin serrate, midrib slightly prominent adaxially, prominent abaxially, secondary veins 8–13 pairs, prominent abaxially, tertiary veins scalariform-reticulate, distinct abaxially; petioles 1–5 cm long, glabrous. Inflorescences terminal, or occasionally terminal and axillary on the upper stem, a corymbose cyme, 6–18 cm in diameter; bract and bracteoles leafy or narrowly lanceolate to linear, persistent. Sterile flowers (functional male flowers) with sepals 3, rarely 2 or 4, white, rarely shallowly tinged with pink, ovate, broadly ovate, suborbicular, 0.4–1.4 × 0.4–1.4 cm, apex obtuse to rounded. Fertile flowers hermaphrodite, pedicellate; pedicels 0.3–1.3 cm long, puberulous. Calyx tube cupular, 1.2–1.8 mm long, puberulous; lobes broadly triangular-ovate, 0.8–1.2 mm long, puberulous, apex rounded, margin ciliolate. Petals white, rarely shallowly tinged with pink, elliptic to suborbicular, 3.7–4.5 mm long. Stamens 28–32, 2.8–5.2 mm long, anthers 0.6–0.8 mm long, filaments 2.2–4.4 mm long, white, glabrous, flattened. Ovary fused with calyx tube, 3-locular, 22–31 ovules per locule. Styles 3, 0.9–1.1 mm long in anthesis, elongate to 1.2–1.8 mm in fruiting. Capsules ellipsoid to subglobose, 2.5–3.8 mm long, 2.2–3.5 mm in diameter. Seeds brown, 1.1–1.5 mm long (including wings); wings translucent and lighter than the seed body color.

##### Distribution.

Japan, Kagoshima Prefecture, Kimotsuki Mountains in the Osumi Peninsula (Fig. 1).

##### Habitat and ecology.

**Figure 3.**
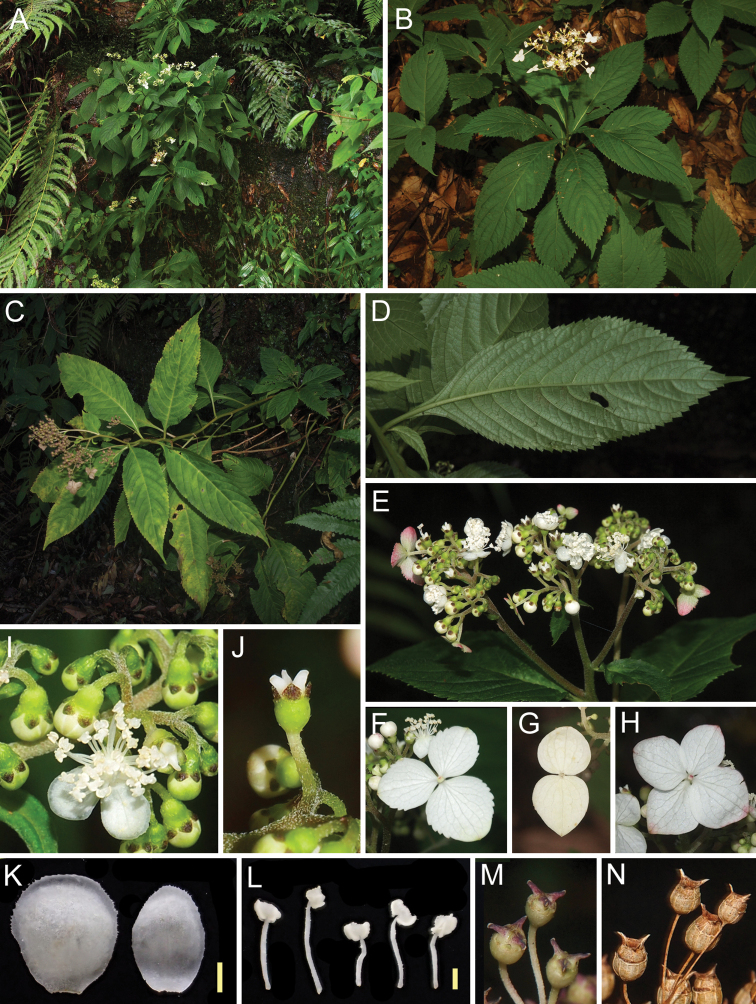
*Hydrangeamarunoi* Tagane & S. Fujii, sp. nov. **A–C** habit **D** abaxial lower leaf surface **E** inflorescence **F–H** petaloid calyx lobes in marginal flower **I** flower and flower buds **J** calyx and styles after anthesis (petals and anthers fallen) **K** petals **L** stamens **M** fruits **N** dried fruits. Scale bars: 1 mm (**K, L**).

*Hydrangeamarunoi* usually grows on semi-shaded wet rocks along streams (Fig. 3A, C), where it grows with *Hymenaspleniummurakami-hatanakae* Nakaike (Aspleniaceae), *Leptochilusneopothifolius* Nakaike (Polypodiaceae), *Thelypterisesquirolii* (Christ) Ching (Thelypteridaceae), T.pozoi (Lag.) C.V.Morton subsp. mollissima (Fisch. ex Kunze) C.V.Morton, *Tricyrtisaffinis* Makino (Liliaceae), *Ophiorrhizajaponica* Blume (Rubiaceae), and *Pileahamaoi* Makino (Urticaceae). Only one soil-growing population was identified on the steep slope of the valley near the Mt. Nokubidake summit (897 m elevation) (Fig. 3B). Flowering specimens were collected from August to September, and fruiting specimens were collected from late September to December.

##### Etymology.

The species epithet *marunoi* is named after Mr. Katsutoshi Maruno, a local botanist who made significant contributions, including elucidating the flora of Kagoshima Prefecture and collecting specimens of this species, as cited earlier.

##### Vernacular name.

Kimotsuki kusa-ajisai (suggested here). ‘Kimotsuki’ named after the Kimotsuki Mountains in Osumi Peninsula where the species occur and ‘kusa-ajisai’ is the common Japanese name for the species of Hydrangeasect.Cardiandra.

##### Conservation status.

Vulnerable (VU). *Hydrangeamarunoi* is known from several populations in Osumi Peninsula (Fig. 1) and the total number of individuals is estimated to be fewer than 1000. Based on the specimen records, the extent of occurrence (EOO) is calculated to be 162 km^2^ by GeoCAT (Bachman et al. 2011) and the area of occupancy (AOO) is 40 km^2^. Some of the habitats are located within the protected area of the Kirishima-Kinkowan National Park and the Inaodake Nature Conservation Area, and the habitat has not been disturbed. Given this situation, it is assessed here as Vulnerable according to the IUCN criterion D (IUCN 2012, 2019).

##### Notes.

The style length is one of the critical characteristics in delimiting the taxa of the Japanese Hydrangeasect.Cardiandra (Ohba 1985a, b, 2001; Ohashi 2017). Ohashi (2017) described the *Hydrangeaalternifolia* style length as 1–1.5 mm (fruiting), whereas Ohba (1985b, 2001) described it as 0.5–1(–1.2) mm. Our examination of the *H.alternifolia* specimens resulted in the style length varied from 0.6–1.2 mm, supporting Ohba’s description. One possible explanation is that Ohashi (2017) regarded *H.marunoi* as an infraspecific variation of *H.alternifolia*, and the length of 1–1.5 mm might include the range of *H.marunoi*.

*Hydrangeamarunoi* is endemic to the Kimotsuki Mountains of the Osumi Peninsula, located in the southernmost part of Kyushu Island. Other taxa endemic to this area include Rhododendronmayebarae Nakai et H. Hara var. ohsumiense T. Yamaz. (Ericaceae; Yamazaki 1984), R.yakumontanum (T. Yamaz.) T. Yamaz. var. katsumarunoanum Minamitani (Ericaceae; Minamitani et al. 2018), and *Lysimachiaohsumiensis* H. Hara (Primulaceae Hara & Kurosawa, 1959). Further research in this region may reveal new taxa.

##### Additional specimens examined.

Japan. Kagoshima Pref. Kimotsuki Town: Kishiragoe, 12 Aug. 1916, fl., *Z. Tashiro s.n.* (TNS 28658); Mankuro, 9 Sept. 2008, fl., *K. Maruno s.n.* (KAG088530); ibid., 10 Sept. 2008, fl., *K. Maruno s.n.* (KAG 088557); Mt. Hoyoshi, 10 Sept. 2008, fl., *K. Maruno s.n.* (KAG 088561); ibid., 27 Oct. 2008, fl., *K. Maruno s.n.* (KAG 088641); Uchinoura, Samuta Forest Road, 6 Sept. 2009, ster. with last year’s infr., *K. Maruno s.n.* (KAG 088825); Uchinoura, 3 Aug. 1946, fl., *I. Furusawa s.n.* (TI); along Oda River, 15 Sept. 2009, fl., *K. Maruno s.n* (KAG 088889, KAG 088890, KAG 088891, KAG 088892, KAG 088893, KAG 088894); ibid., 8 Aug. 2021, fl., *S. Fujii 19274* (KYO, TI, TNS), *19280* (KYO, TI, TNS); ibid., 8 Aug. 2021, fl., *S. Tagane K1633* (KAG 153194), *K1638* (KAG 153199); ibid., 4 Dec. 2021, fr., *S. Tagane & K. Fu*se *K1828* (KAG 153605). Kinko Town: Mt. Uodake (Mt. Hassan), 15 Sept. 1988, fl., *K. Maruno s.n.* (KAG 156990); ibid., 25 Sept. 1988, young fr., *K. Maruno s.n.* (KAG 082268); Mt. Karekidake, 16 Sept. 2009, fl., *K. Maruno s.n.* (KAG088911); Mt. Aranishi, 5 Nov. 2009, young infl., *K. Maruno s.n.* (KAG 088956); Minamiosumi Town: Satahetsuka, 2 Sept. 2004, fl., *Y. Morita & K. Maruno s.n.* (KAP 00400106s, KAP 00400107s,); Mt. Nokubidake, fl. bud, 9 Aug. 2008, *K. Maruno s.n.* (KAG 088497); ibid., fl. bud, 9 Aug. 2008, *T. Ohya s.n.* (KAP 00800421s, KAP00800443s); ibid., 29 Sept. 2021, fl., *S. Tagane K1658* (KYO), *K1659* (TI), *K1660* (KAG 153218) *& K1661* (TNS).

**Figure 4.**
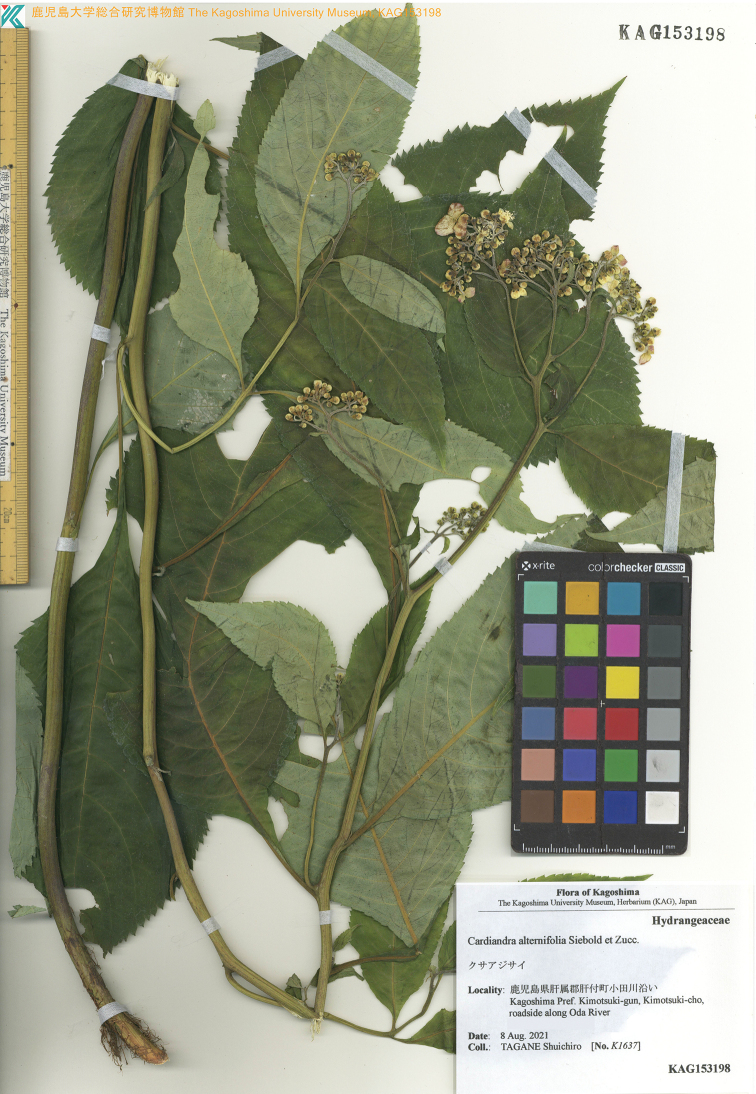
Holotype of *Hydrangeamarunoi* Tagane & S. Fujii, sp. nov. (*S. Tagane K1637* [KAG 153198]).

## ﻿Discussion

In appearance, *H.marunoi* is more similar to *H.alternifolia*, typically having three-petaloid calyx lobes in marginal flowers, than to *H.moellendorffii*, which has two-petaloid calyx lobes or to *H.amamiohsimensis* without petaloid calyx lobes. However, the MIG-seq tree (Fig. 2) clearly exhibited that *H.marunoi* is more closely related to the clade consisting of *H.amamiohsimensis* endemic to Amami-Oshima, an island located 583 km south of Kyushu Island, and *H.moellendorffii* of Iriomote Island, located 282 km east of Taiwan, than to *H.alternifolia* widely distributed on Honshu, Shikoku, and Kyushu islands (Fig. 2). The three samples K1652–1654 collected from Mt. Takakuma in the northern Osumi Peninsula, just 30 km north and the closest to the collection site of *H.marunoi* among the collection sites of *H.alternifolia* in this study, are genetically divergent from *H.marunoi*. The flowers of individuals from Mt. Takakuma showed 26 stamens and 1.1–1.2 mm long styles, which are typical characters of *H.alternifolia*. The habitat preference also supported this relationship; *H.marunoi* mostly grows on wet rocks by the stream, which is the typical habitat of *H.amamiohsimensis* on Amami-Oshima and *H.moellendorffii* on Iriomote Island, whereas *H.alternifolia* usually grows on the soil in the forest.

The MIG-seq tree also revealed that a clade *H.alternifolia* consisting of three samples JPN10093, 10106 and 10107, collected from the Sea of Japan (western) side of Honshu Island (yellow squares in Fig. 1, designated as clade A) is highly differentiated from the rest of *H.alternifolia* samples (designated as *H.alternifolias.str.*) (Fig. 2). Further morphological studies based on additional materials are required to characterize this clade.

### ﻿Key to the species of Hydrangeasect.Cardiandra (partly based on Ohba 1985b; Wei and Bartholomew 2001; Ohashi 2017)

**Table:** 

1	Leaves sparsely scattered along stem, alternate	**2**
–	Leaves distally on stem, usually 4–8 fascicled [China (Zhejiang), Taiwan]	***H.densifolia***
2	Petaloid calyx lobes in marginal flowers present	**3**
–	Petaloid calyx lobes in marginal flowers absent [Japan (Amami-Oshima Island)]	***H.amamiohsimensis***
3	Petaloid calyx lobes in marginal flowers 3 (rarely 2 or 4) [Japan (Honshu, Shikoku, Kyushu)]	**4**
–	Petaloid calyx lobes in marginal flowers 2 (very rarely 3) [China, Japan (Iriomote Island)]	***H.moellendorffii***
4	Stamens 15–26; styles to 1.2 mm long in fruiting; seeds 0.7–1 mm long [Japan (Honshu, Shikoku, Kyushu)]	***H.alternifolia***
–	Stamens 28–32; styles 1.2–1.8 mm long in fruiting; seeds 1.2–1.5 mm long [Japan (Kyushu: Kimotsuki Mountains)]	***H.marunoi***

## Supplementary Material

XML Treatment for Hydrangea marunoi

## Supplementary materials

Supplementary material 1 Table S1 Data type sample list Explanation note Sample list used for phylogenetic analysis in this study. https://binary.pensoft.net/file/755439This dataset is made available under the Open Database License (http://opendatacommons.org/licenses/odbl/1.0/). The Open Database License (ODbL) is a license agreement intended to allow users to freely share, modify, and use this Dataset while maintaining this same freedom for others, provided that the original source and author(s) are credited.Shuichiro Tagane, Shinji Fujii, Shun K. Hirota, Akiyo Naiki, Tetsukazu Yahara
