# Supplementary material for: ﻿ Hydrangeamarunoi (Hydrangeaceae), a new species from Osumi Peninsula, southern Japan
Source: PhytoKeys. 2022 Oct 11;211:33–44. doi: 10.3897/phytokeys.211.89452 (PMC9836688; doi:10.3897/phytokeys.211.89452)
Supplement: Supplementary material 1 — Table S1 [file phytokeys-211-033_article-89452__-s001.docx]

**Supplementary Table 1.** Sample list used for phylogenetic analysis in this study.

| **Taxon** | **Sample number** | **Prefecture** | **Latitude and Longitude (degree)** | **Date collected** |
| --- | --- | --- | --- | --- |
| *Hydrangea alternifolia* | HT4615 | Yamaguchi | 34.463 N 132.057 E | 2021-08-30 |
|  | HT4616 | Yamaguchi | 34.462 N 132.058 E | 2021-08-30 |
|  | HT4617 | Yamaguchi | 34.461 N 132.059 E | 2021-08-30 |
|  | HT4618 | Yamaguchi | 34.460 N 132.059 E | 2021-08-30 |
|  | HT4619 | Yamaguchi | 34.458 N 132.061 E | 2021-08-30 |
|  | JPN1121 | Kagoshima | 31.848 N 130.738 E | 2020-08-10 |
|  | JPN2693 | Aichi | 34.939 N 137.399 E | 2020-10-14 |
|  | JPN2872 | Wakayama | 33.785 N 135.896 E | 2020-10-27 |
|  | JPN2876 | Wakayama | 33.785 N 135.896 E | 2020-10-27 |
|  | JPN6547 | Kochi | 33.780 N 134.019 E | 2021-06-25 |
|  | JPN10091 | Osaka | 34.426 N 135.659 E | 2020-07-13 |
|  | JPN10093 | Niigata | 36.848 N 137.829 E | 2021-12-21 |
|  | JPN10094 | Osaka | 34.345 N 35.411 E | 2021-11-05 |
|  | JPN10095 | Shizuoka | 34.806 N 137.940 E | 2021-10-29 |
|  | JPN10096 | Hiroshima | 35.042 N 132.854 E | 2021-10-15 |
|  | JPN10097 | Gifu | 35.546 N 137.101 E | 2021-10-10 |
|  | JPN10098 | Kochi | 33.715 N 133.310 E | 2021-10-02 |
|  | JPN10099 | Kochi | 33.778 N 133.557 E | 2021-10-01 |
|  | JPN10100 | Kochi | 35.130 N 137.719 E | 2021-08-20 |
|  | JPN10101 | Kagoshima | 31.780 N 130.928 E | 2021-08-10 |
|  | JPN10102 | Kagoshima | 31.779 N 130.935 E | 2021-08-10 |
|  | JPN10103 | Kagoshima | 31.265 N 131.051 E | 2021-10-05 |
|  | JPN10104 | Osaka | 34.426 N 135.659 E | 2021-10-05 |
|  | JPN10105 | Chiba | 35.324 N 140.158 E | 2021-10-05 |
|  | JPN10106 | Tottori | 35.478 N 134.403 E | 2021-10-05 |
|  | JPN10107 | Kyoto | 35.35 N 135.764 E | 2021-12-21 |
|  | K384 | Mie | - | 2020-09-12 |
|  | K1623 | Nagasaki | 32.996 N 130.072 E | 2021-08-01 |
|  | K1652 | Kagoshima | 31.527 N 130.793 E | 2021-08-29 |
|  | K1653 | Kagoshima | 31.527 N 130.793 E | 2021-08-29 |
|  | K1654 | Kagoshima | 31.527 N 130.793 E | 2021-08-29 |
|  | K1662 | Shimane | 35.198 N 132.795 E | 2021-09-01 |
|  | K1663 | Shimane | 35.198 N 132.795 E | 2021-09-01 |
|  | K1664 | Shimane | 35.198 N 132.795 E | 2021-09-01 |
|  | K1665 | Shimane | 35.198 N 132.795 E | 2021-09-01 |
|  | TG01014 | Kumamoto | 32.190 N 130.613 E | 2019-09-14 |
| *Hydrangea alternifolia* var. *hakonensis* | JPN10092 | Kanagawa | 35.162 N 139.076 E | 2021-12-21 |
| *Hydrangea amamiohsimensis* | KAG159893 | Kagoshima | - | - |
|  | KAG159894 | Kagoshima | - | - |
| *Hydrangea bifida* | JPN4970 | Saitama | 36.045 N 138.864 E | 2021-05-11 |
| *Hydrangea marunoi* | JPN9950 | Kagoshima | - | 2021-12-04 |
|  | K1633 | Kagoshima | - | 2021-08-08 |
|  | K1637 | Kagoshima | - | 2021-08-08 |
|  | K1638 | Kagoshima | - | 2021-08-08 |
|  | K1658 | Kagoshima | - | 2021-08-29 |
|  | K1659 | Kagoshima | - | 2021-08-29 |
|  | K1660 | Kagoshima | - | 2021-08-29 |
|  | K1661 | Kagoshima | - | 2021-08-29 |
|  | KAG088891 | Kagoshima | - | 2009-09-15 |
| *Hydrangea moellendorffii* | K945 | Okinawa | - | 2020-11-14 |
|  | JPN10143 | Okinawa | - | 2018-03-04 |
|  | JPN10144 | Okinawa | - | 2019-10-30 |
|  | JPN10145 | Okinawa | - | 2021-02-14 |
